# Resection of the Four External Fifths of the Clavicle

**Published:** 1846-11

**Authors:** 


*Resection of the four external fifths of the Clavicle.* By M. Chaumet.—Marie Clermontel, æt. 18, was admitted into the Hôpital St. André of Bourdeaux, June 30, 1845. Nine months previously she expressed vague pains in the right clavicle. A tumour soon commenced which has attained the size of the shut hand; it is immoveable, hard, mammillated, and occupies nearly the four external fifths of the clavicle: it is the seat of moderate pain, and does not impede the motions of the right arm, which is not, however, quite so strong as the opposite one. July 15th M. Chaumet laid the tumour bare by a semi-elliptical incision, convex anteriorly and inferiorly, which extended from within half an inch of the sterno-clavicular articulation to the summit of the acromion. After dissecting up the flap, a portion of which was removed, as its size would have interfered with coaptation of the parts after the removal of the tumour, a vertical incision was made at the sternal extremity of the first along the inner border of the sterno-mastoid muscle. The tumour was then detached from its connections without its being necessary to tie a single artery. The clavicle was now divided at its internal fourth with the chain-saw, and the coraco-clavicular ligaments being cut, the bone was removed. Three points of suture were applied, and to diminish the extent of the cicatrix, the shoulder was approximated to the residue of the clavicle by a circular bandage. Irrigation with cold water was commenced two hours after the operation, and continued till the tenth day. On the 54th day the remains of the clavicle was slightly elevated by the sterno-mastoid muscle. On the 70th day the girl returned home, enjoying slight motion of the arm.—*Ibid, from Journ. de Med. de Bourdeaux.*

